# Identification of Therapeutic Candidates for Chronic Lymphocytic Leukemia from a Library of Approved Drugs

**DOI:** 10.1371/journal.pone.0075252

**Published:** 2013-09-20

**Authors:** Min Shen, Yaqin Zhang, Nakhle Saba, Christopher P. Austin, Adrian Wiestner, Douglas S. Auld

**Affiliations:** 1 National Center for Advancing Translational Sciences, National Institutes of Health, Rockville, Maryland, United States of America; 2 National Heart, Lung, and Blood Institute, National Institutes of Health, Bethesda, Maryland, United States of America; Bioinformatics Institute, Singapore

## Abstract

Chronic lymphocytic leukemia (CLL) is an adult lymphoid malignancy with a variable clinical course. There is considerable interest in the identification of new treatments, as most current approaches are not curative. While most patients respond to initial chemotherapy, relapsed disease is often resistant to the drugs commonly used in CLL and patients are left with limited therapeutic options. In this study, we used a luminescent cell viability assay based on ATP levels to find compounds that were potent and efficacious in killing CLL cells. We employed an in-house process of quantitative high throughput screening (qHTS) to assess 8 concentrations of each member of a 2,816 compound library (including FDA-approved drugs and those known to be bio-active from commercial suppliers). Using qHTS we generated potency values on each compound in lymphocytes donated from each of six individuals with CLL and five unaffected individuals. We found 102 compounds efficacious against cells from all six individuals with CLL (“consensus” drugs) with five of these showing low or no activity on lymphocytes from a majority of normal donors, suggesting some degree of specificity for the leukemic cells. To our knowledge, this is the first study to screen a drug library against primary CLL cells to identify candidate agents for anti-cancer therapy. The results presented here offer possibilities for the development of novel drug candidates for therapeutic uses to treat CLL and other diseases.

## Introduction

Chronic Lymphocytic Leukemia (CLL), the most common leukemia in the Western world, is characterized by the accumulation of monoclonal CD5+ mature B cells in the peripheral blood (PB), lymph nodes (LN) and bone marrow (BM). The majority of cases are diagnosed in asymptomatic patients with an incidental finding of lymphocytosis or lymphadenopathy [1]. The standard of care for CLL is watchful waiting of asymptomatic patients and chemoimmunotherapy for patients with active disease [2]. This clinical approach to CLL is guided by the absence of a curative chemotherapy regimen, the results of clinical trials that have shown no benefit for early chemotherapy in asymptomatic patients, and the relatively long natural history of the disease with a median survival of 11 years [3]. CLL is divided into two main subgroups based on the presence or absence of acquired somatic mutations in the immunoglobulin heavy-chain variable region (IGHV) expressed by the leukemic B cells. Patients with mutated IGHV have a more indolent disease and longer overall survival than patients whose tumors express an unmutated IGHV gene. High expression of ZAP70 and CD38 are additional markers indicating more rapid disease progression [4]. Cytogenetic alterations are also strong predictors of outcome. In particular, deletion of TP53 locus on 17p and deletion of the ATM locus on 11q are associated with more rapidly progressive disease and inferior response to chemotherapy. Increasingly, risk stratified treatment approaches are pursued for patients with these adverse prognostic markers [5,6].

Over the past 20 years, therapy for CLL has improved dramatically [7]. The frequency of complete responses achieved with traditional therapy using oral chlorambucil (single-agent alkylator) in the treated patients was less than 5%, while modern regimens using multi-agent chemoimmunotherapy can reliably produce complete responses in over 50% of patients. This notable improvement is primarily attributable to an increase in the number and activity of therapeutic agents recently made available to treat CLL, such as fludarabine [8,9], a purine analogue-based chemotherapy agent as well as monoclonal antibodies rituximab [10] and alemtuzumab [11]. Novel combinations of these agents have emerged as effective new therapies for previously untreated patients. Clinical studies indicate that such combinations can induce higher response rates (including complete responses) than single-agent therapy [12,13]. Those patients who achieve a complete response have superior progression-free survival compared with those who achieve only a partial response. However, there is still considerable interest in identifying new treatments as most current approaches are not curative. While most patients respond to initial chemotherapy, relapse is commonly observed in CLL patients. Relapsed CLL patients are then left with limited therapeutic options. In addition, many challenges remain, such as finding less toxic and equally efficacious regimens for older patients, who are the majority of the population with this disease but may not tolerate some of the more aggressive combination chemoimmunotherapy regimens [1].

In the last decade, several efforts have shown that low molecular weight compounds which have been approved for as drugs can be “repurposed” for new indications, and studied to determine the mechanisms of both beneficial and adverse effects [14-19]. To rapidly and efficiently identify currently FDA approved drugs with anti-CLL activity, we screened approximately 2,800 drugs from the NIH Chemical Genomics Center Pharmaceutical Collection (NPC) [20] against primary CLL cells using a cell viability assay. We utilized a quantitative high-throughput screening (qHTS) format and identified several small molecule drugs that induced significant cytotoxicity in CLL cells with no or little effect on lymphocytes from normal donors, suggesting some degree of specificity for the leukemic cells. As we know that one of the biggest issues with the current chemotherapeutic agents is their cumulative *toxic* effects and lack of specificity, the results presented in this paper provide a new approach that can lead to the discovery of selective chemotherapeutic agents with an improved therapeutic window, and provide a paradigm that can be applied broadly to maximize appropriate uses for currently approved drugs.

## Materials and Methods

### Cell apheresis and culture conditions

Lymphocytes from 13 CLL treatment naïve patients (Table **1**) and 5 normal donors were obtained by lymphapheresis with written informed consent and isolated by gradient centrifugation using Lymphocyte Separation Medium (MP Biomedicals, Solon, OH) and used fresh or cryopreserved in liquid nitrogen in 10% dimethyl sulfoxide, 90% FCS. CLL patients were consented on the institutional review board (IRB) approved at the National Heart, Lung, and Blood Institute (NHLBI, Office of Clinical Affairs, Bldg 10, CRC. Room 6-5140, Bethesda, Maryland 20892-1608) with protocol 04-H-0012 and samples from normal donors were collected by the Department of Transfusion Medicine, National Institutes of Health (NIH). This study was approved by the IRB mentioned above. Fresh cells were temporally placed on ice and plated for screening within 3 hrs of apheresis. Frozen cells were thawed the night before the assay and incubated in T175 flask in AIM V medium to recover overnight. Analysis of IGHV gene status was performed as described [21]. CLL samples were cultured in AIM V serum free medium (Gibco-Invitrogen, Long Island, NY).

**Table 1:** Patient characteristics.

**Study#**	**Gender/Age (y**)	**# prior regimens**	**ALC (x10^9^/L**)	**Rai stage**	**CLL subtype by IGHV**	**CD38%**	**FISH**	**Cells**
**4789**	M/65	0	81.096	2	U	Neg	11q;+12	frozen
**4808**	M/53	0	74.814	4	U	Neg	13q	frozen
**4811**	M/53	0	22.733	4	U	88.3	13q	frozen
**4399**	F/55	0	34.216	2	U	Neg	13q	frozen
**4705**	M/59	0	49.632	4	M	Neg	17p	frozen
**4765**	M/64	0	22.138	2	M	6	13q	frozen
**5476**	F/55	0	90.91	3	U	81	+12	fresh
**5477**	F/49	0	290.92	3	M	0.3	13q	fresh
**5478**	F/57	0	128.7	3	U	43	13q	fresh
**5491**	M/50	0	10.45	1	ND	18	11q; 13q	fresh
**5492**	M/77	0	47.5	2	U	64	+12; 13q	fresh
**5493**	M/58	0	41.48	4	M	Neg	13q	fresh
**5494**	F/78	0	220.94	3	M	90	11q; 13q	fresh

All patients are treatment naïve. Nd = not done.

FISH results denote chromosomal deletion 11q, 13q, 17p, and trisomy 12 (+12).

### NIH Chemical Genomic Center (NCGC) pharmaceutical library

The NCGC pharmaceutical collection (NPC collection) was constructed in house [20]. The comprehensive library at the time of this study contained 2,816 clinically approved and pharmacologically active small molecules, 52% of which are drugs approved by Food and Drug Administration (FDA) for human or animal use in the United States. The rest of the molecules are either approved for human use in other countries (such as Europe, Canada, or Japan) but not approved by the U.S. FDA, or are investigational compounds that have been tested in clinical trials. Additional detailed information on the drugs can be found at http://tripod.nih.gov/npc/.

As the NPC library was prepared for laboratory-based *in vitro* assay screening applications, the following three categories of compounds were excluded in the collection: (1) large molecules with MW > 1,500, such as proteins and antibodies; (2) molecules that are either insoluble in dimethyl sulfoxide (DMSO, a frequently used solvent) or unstable at room temperature; (3) molecules that contain less than 16 atoms, or had no carbon or nitrogen atoms. For use in the qHTS assays, the NPC library was first prepared in either 96 or 384-well plates in DMSO stock with the stock concentrations of the test compounds ranging from 10 mM to 0.13 µM. Then the compounds were serially diluted using a 1:2.236 dilution factor and transferred to 1536-well plates using an Evolution P^3^ system (PerkinElmer Life and Analytical Sciences, Waltham, MA) to make 15 compound plates in inter-plate titration fashion. On each plate the compounds were distributed from column 4 to 48, leaving the first 4 columns to be DMSO only for assay positive controls and concentration-response titration of controls. During screening, the compound plates were sealed and kept at room temperature for up to 6 months, whereas other copies were stored at −80°C for future use.

### Quantitative high-throughput screening (qHTS) and cell viability assay

Compound formatting and qHTS were performed as described previously [22]. The final concentration of the NPC compounds in the 4 µL assay volume ranged from 57 µM to 0.7 nM in 1:5 dilution steps. The positive control plate format was as follows: columns 1 and 3, DMSO only; column 2, doxorubicin, a known cytotoxic chemotherapy agent in a 1:2 dilution series from 10 µM to 5 nM in DMSO; and column 4, doxorubicin at 10 µM in DMSO.

Cell viability was measured using a luciferase-based ATP quantitation assay (CellTiter-Glo^TM^, Promega). The intracellular ATP content indicates the number of viable (i.e. metabolically competent) cells after compound treatment. Four µL of CLL cells resuspended in AIM medium at 1,330,000 cells/ml, were dispensed into each well of white, solid bottom, 1536-well tissue culture–treated plates using a Multidrop-Combi dispenser and incubated overnight. After that, a total of 23 nL of compounds at 8 selected concentrations from the NPC library or positive control (10 mM stock of doxorubicin hydrochloride) in DMSO was transferred to each well of the assay plate using a pintool (Kalypsys, San Diego, CA), and the plates were further incubated at 37 °C with 5% CO_2_ for 24 hours to allow the reaction. Then 4µL of CellTilter-Glo luminescent substrate mix was added to each well. The plate was incubated at room temperature for 15 minutes. The plates were measured on a ViewLux plate reader (PerkinElmer) with clear filter. The final duration of incubation was based on the results of assay optimization experiments demonstrating that there was no significant difference in endpoint readouts between 24, 48 and 72-hour time points. DMSO tolerance experiments with each primary cell of CLL patients or normal donors showed no effect on viability at concentrations up to 0.6%. The test volume of 5,000 cells/4 μL/well was selected as the final assay condition in cell density tests based on the assay performance statistics: signal/background ratio, coefficient of variation and Z’-factor (data not shown).

In the confirmation study, fresh stocks of selected active compounds were prepared from powder and re-plated within a single 1536-well plate. These compounds were then re-tested in the cell viability assay using 12 point titrations with concentration ranging from 57 µM to 0.3 nM with 1:3 dilution.

### Caspase-3/7, caspase-8 and caspase-9 activation assay

The effect of selected compounds on caspase activity was measured using a homogeneous luminescent method (Caspase-Glo® 3/7, 8 and 9 kits, Promega, Madison, WI). Briefly in this assay, caspase induced by cells cleaves a pro-luciferin substrate where a tetrapeptide caspase substrate - DEVD (asp-glu-val-asp) is cleaved to free aminoluciferin, which can be used as a substrate by luciferase yielding a bioluminescent signal. The luminescent signal is proportional to the amount of caspase activity present in the cells [23]. Cells were dispensed in culture medium at 5,000/cells/5uL/well in 1536-well white/solid-bottom assay plates. The cells were incubated for overnight at 37°C. The compounds (23nL/well) were added via the pin tool. The treated cells were further incubated for 24 h at 37°C, followed by the addition of the Caspase-Glo 3/7, 8 or 9 reagents at 5uL/well. After a 30 min incubation at room temperature, the luminescence intensity of the assay plates was measured using a ViewLux Plate Reader. The caspase activity was normalized to DMSO control.

### Data analysis and clustering of compounds by activity outcomes

To determine compound activity in the qHTS assay, the concentration-response data for each sample was plotted and modeled by a four parameter logistic fit yielding IC_50_ and efficacy (maximal response) values as previously described [22]. Data normalization and curve fitting were performed using in-house informatics tools. Briefly, raw plate reads for each titration point were first normalized relative to the positive control compound (100%) and DMSO-only wells (basal, 0%), and then corrected by applying a pattern correction algorithm using compound-free control plates (i.e., DMSO-only plates) at the beginning and end of the NPC compound plate stack. Compounds were designated as Class 1–4 according to the type of concentration-response curve (CRC) observed [22]. Usually the qHTS screen yielded hits with a wide range of potencies and with substantial variation in the quality of the corresponding CRCs (efficacy and number of asymptotes), which included samples associated with shallow curves or single-point extrapolated concentration responses; these were assigned as low-confidence actives. In brief, Class 1.1 and 1.2 were the highest-confidence complete CRCs containing upper and lower asymptotes with efficacies ≥ 80% and < 80%, respectively. Class 2.1 and 2.2 were incomplete CRCs having only one asymptote with efficacy ≥ 80% and < 80%, respectively. Class 3 CRCs showed activity at only the highest concentration or were poorly fit. Class 4 CRCs were inactive having a curve-fit of insufficient efficacy or lacking a fit altogether.

Compounds from the primary qHTS screen were further classified into three categories according to the quality of curve fit and efficacy. Actives: compounds in curve class 1.1, 1.2, 2.1 and 2.2 curves with efficacy higher than 60%; inactives: compounds with class 4 curves; inconclusive: all other compounds including those shallow curves and curves with single point extrapolated activity.

Based on the definition of actives, inactives and inconclusive mentioned above, compounds were further clustered hierarchically using Spotfire DecisionSite 8.2 (Spotfire Inc., Cambridge, MA) based on their activity outcomes from the primary screen across a wide variety of cell viability assays against different cell lines or primary patient cells. Each compound was converted into an integer which represents its activity outcome in each cell viability assay. In brief, integer 1 represents compounds in “active” category which is corresponding to red in the heat map. Integer 2 represents compounds in “inconclusive” category which is corresponding to light red in the heat map. If a compound was inactive in an assay, integer 3 was assigned to such category and it was highlighted as white in the heatmap. Compounds not tested in the assay were labeled as 4 and shown as grey in the heat map. Different activity outcomes were observed for each cell viability assay, the compounds were categorized based on the similarity metric derived from the activity profiles.

## Results

### Identification of agents that are toxic to CLL primacy patient cells through pharmacological profiling of drugs

To identify drugs that are cytotoxic for CLL cells we determined the potency of approximately 2,800 NPC library compounds. Initially, primary cells collected from six CLL patients and five unaffected donors were screened in parallel against the NPC library compounds. For the screen we chose a robust assay of cellular viability based on measuring ATP levels with bioluminescence from the ATP-dependent firefly luciferase enzyme. This assay can be readily automated and is also the standard measure of cytotoxicity for many other cell lines in our database allowing for robust comparison of viability data. In a first round of screening frozen cells were used followed by confirmation studies with freshly obtained primary cells from additional patients. Doxorubixin, a widely used chemotherapeutic drug, was used as positive control. The assay performed well across the 132 1536-well plates used to screen the CLL patient samples with a Z’-factor = 0.86 as well as the 110 plates used to screen the normal donor cells with a Z’-factor = 0.76. The signal-to-background ratio was 11.2 in average for CLL screen.

**Figure 1:**
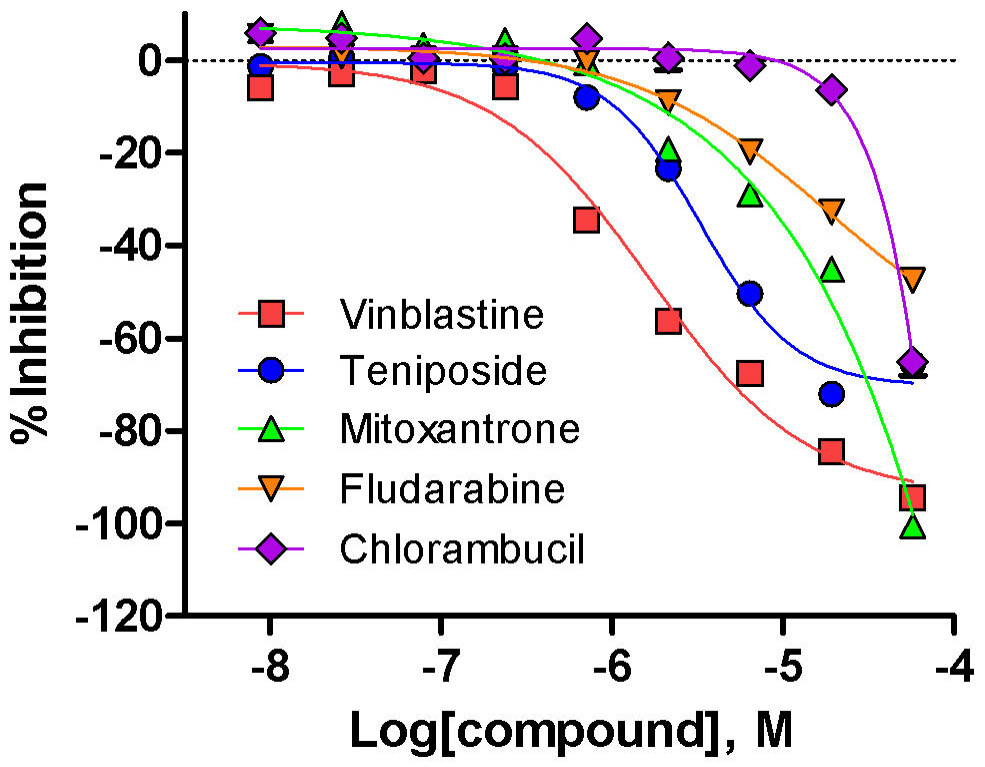
Examples of curve class definition using CLL known drugs or antineoplastic agents. Data was normalized to DMSO basal (0%) and control compound doxorubicin (-100%).

Of the 2,816 compounds tested, 431 showed cytotoxicity in at least one individual CLL patient cell sample. The remaining 2,385 compounds (84.7%) did not induce cytotoxicity in any of the patient cell samples and were classified as inactive. Patient sample IDs #4808 and #4705 represented the most and least sensitive patient cells resulting in 356 and 117 actives in the screen, respectively (Figure **S1**). Here we define actives as those compounds that showed CRC classes of 1.1, 1.2, 2.1 and 2.2 with an efficacy > 60% (see Materials and Methods). As illustrated in Figure **1**, vinblastine and teniposide were showed complete dose-response curves of type 1.1 and 1.2, with efficacy over or below 80% respectively. Similarly, mitoxantrone and fludarabine were showed dose-response curves of type 2.1 and 2.2 with varied efficacy, but their dose-response curves were not complete at the testing concentrations. Chlorambucil only showed significant cell killing at the top concentration, so it was characterized as having curve class 3 activity (Figure **1**). Overall, the results from all six patient cell samples demonstrated differential sensitivity to drug mediated cytotoxicity based on the number of active compounds identified in the viability assay and were ranked as: #4808, 356 (12.6% of compounds active) > #4811, 300 (10.6%) > #4789, 270 (9.6%) > #4399, 168 (6.0%) > #4765, 152 (5.4%) > #4705, 117 (4.2%).

**Figure 2:**
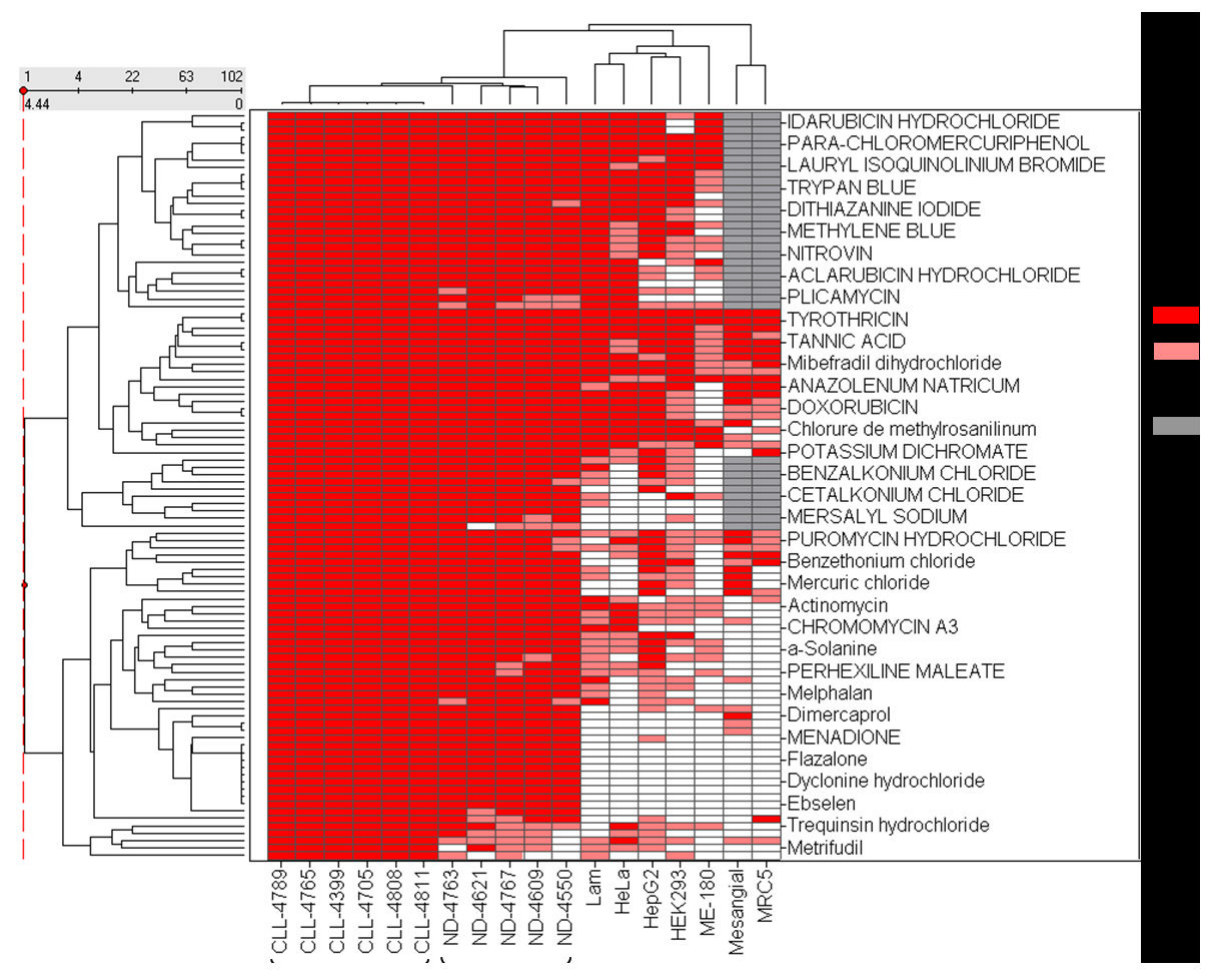
Activity profile of 102 compounds active in CLL primary patient cells compared to normal donor lymphocytes and selected cell lines. Drug names are given at right and the cell types are listed at the bottom of the heat map. Active means compounds in curve class 1.1, 1.2, 2.1 and 2.2 curves with efficacy higher than 60%; Inactive means compounds with class 4 curves; Inconclusive designates all other compounds including those shallow curves and curves with single point extrapolated activity.

A total of 235 compounds showed cytotoxicity in at least three individual CLL preparations and 102 compounds from the primary screen were classified as “consensus” hits which were pan active across all six CLL patient cells on the basis of high confidence curve classes and IC_50_ values <30 µM, Thus, 3.6% of all compounds tested were considered to have anti CLL activity. The activity outcomes and the potency heatmap are shown in Figure **2**.

We were able to identify multiple agents across different therapeutic categories and modes of action that showed potent toxicity to the CLL cells from 102 consensus hits. We selected 41 compounds for further study; 38 compounds with anti-CLL activity and potency less than 10 µM and three drugs (fludarabine, chlorambucil and bendamustine) that are currently used in standard clinical treatment of CLL (Table **S1**). Among the selected compounds, 29 are antineoplastic agents, 5 are antibacterial agents, and others included drugs used for the treatment of hypertension, inflammation, rheumatoid arthritis and heavy metal poisonings.

**Figure 3:**
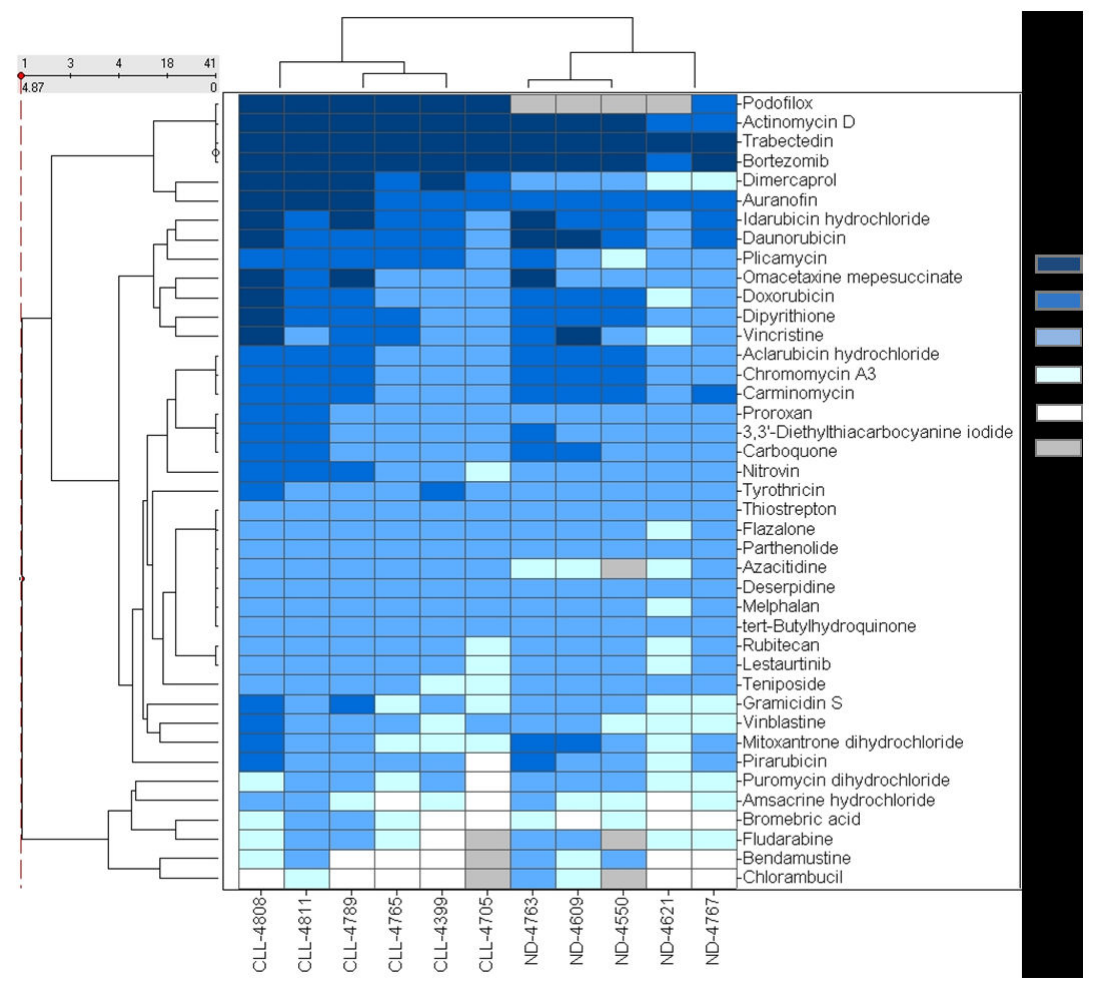
A close-up view of 41 selected compounds with the heat map scaled by the IC_50_ of the compound in CLL cytotoxicity assay. The potency categories are shown in different color. Grey bar represents inactive compound.

The IC_50_ values of the consensus hits ranged from 8.6nM to 26.8µM. In most cases the IC_50_ values were comparable between different patient samples (Figure **3**). For example, trabectedin and bortezomib, produced cytotoxicity in all CLL cell samples at similar concentrations. There were twenty compounds that exhibited average potency values of <1 µM in all CLL preparations; however, the majority of these compounds had potency values ranging from 1 to 10 µM.

In contrast, other compounds such as idarubicin and vinblastine, showed different potencies between cell samples (Figure **3**). The IC_50_ of idarubicin was 0.1 µM against patient #4789 and #4808, yielding a 32-fold potency shift compared to patient #4705, in which the IC_50_ of idarubincin was 3.2 µM. Vinblastine showed a greater potency shift as this compound was >100-fold more potent in CLL sample #4808 than #4399.

### Toxicity profiling of compounds with anti-CLL activity against primary cells from unaffected donors

A drawback of many current chemotherapeutic agents is that these are cytotoxic to both cancer and normal cells. Therefore, we also screened each compound against lymphocytes donated from 5 healthy individuals. The CRC classification, potency, and efficacy were determined for the entire collection and these values were used to evaluate selective cytotoxicity between CLL and normal lymphocyte samples. Of the 102 compounds that were cytotoxic to all 6 CLL samples, 96 (94.1%) were also cytotoxic to cells derived from normal donors at comparable potency (Figure **2**).

Only 5 compounds showed differential cytotoxicity (e.g either differential efficacy or a considerable IC_50_ shift, >5-fold) between CLL cells and lymphocytes from normal donors, suggesting some degree of specificity against the leukemic cells. These were auranofin, azacitidine, dimercaprol, plicamycin, and podofilox.

### Toxicity profiling of CLL consensus hits in several normal and solid tumor cell lines

To test whether the consensus drugs identified from the screening have specific anti-cancer activity to CLL, several other cell viability assays employing other types of cancer and transformed lines that had been screened against the NPC drug library were compared to the CLL viability results. In general a distinct activity pattern was observed for CLL cells however some CLL consensus drugs showed similar toxicity in other normal and solid tumor cell lines (Figure **2**). These included MRC-5 human fetal lung fibroblasts [24], human kidney glomerular mesangial cell line, ME-180 human cervical carcinoma cell line, HEK293 human embryonic kidney 293 cell line, HepG2 human liver carcinoma cell line, Hela human cervical cancer cell line, and a Lymphangioleiomyomatosis (LAM, a rare lung disease that results in a proliferation of disorderly smooth muscle growth) cell line. For example, the antibiotic tyrothricin was pan-active in the cytotoxicity profiling assays (potency varied <5-fold). Specifically, many drugs that showed a strong anti-proliferative effect in CLL cells showed none or reduced cytotoxicity in other cancer cell types. In addition, the 102 CLL consensus drugs were found to be highly selective to ME-180 cervical cancer cells in which only 7 drugs showed prominent cytotoxicity including trypan blue, ivermectin, phenylmercuric acetate, tyrothricin, sanguinarine, tomatine and lissamine green B. Taken together this demonstrates a specific toxicity profile for the CLL viability assay with few generally cytotoxic compounds identified.

### Data agreement and assay reproducibility using fresh and frozen cells

To confirm compound activity, fresh samples of these compounds were prepared from powder sources re-plated within one 1536-well plate and re-rested in the cell viability assay using 12 point titrations covering a concentration range from 0.3 nM to 57 µM. Fresh primary cells were also obtained from two unaffected normal donors and seven CLL patients whose blood samples were collected within hours of apheresis and which had not been included in our previous experiments.

**Figure 4:**
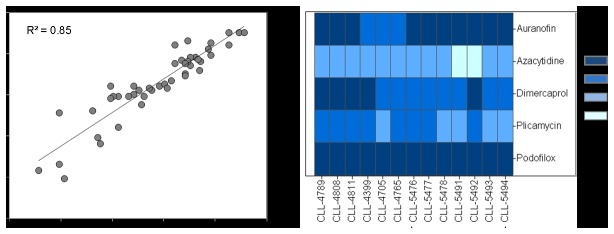
Data agreement and assay reproducibility using CLL primary patient cells from fresh and frozen samples. **A**. Potency correlation between data obtained from fresh cells and frozen cells for 41 selected compounds in follow-up studies. **B**. Heatmap of potency correlation for five selected drugs: auranofin, azacitidine, dimercaprol, plicamycin, and podofilox. The potency categories are shown in different color.

The follow-up plate containing 41 selected compounds was tested against the fresh samples under the same assay conditions. Activity was confirmed in all retested compounds, yielding a confirmation rate of 100%. Sixteen had IC_50_ values < 1 µM, 10 of which were antineoplastic agents including aclarubicin hydrochloride, actinomycin D, bortezomib, chromomycin A3, idarubicin hydrochloride, omacetaxine mepesuccinate, plicamycin, trabectedin, vinblastine and vincristine (see Table **S1**). Comparing the potency (LogIC_50_) between fresh CLL samples and frozen cell experiments showed excellent concordance with r^2^ > 0.8 (Figure **4A**). All five compounds that showed differential cytotoxicity between CLL cells and lymphocytes from normal donors in the screening were validated with similar potency and efficacy in fresh CLL patient lymphocytes (Figure **4B**).

**Figure 5:**
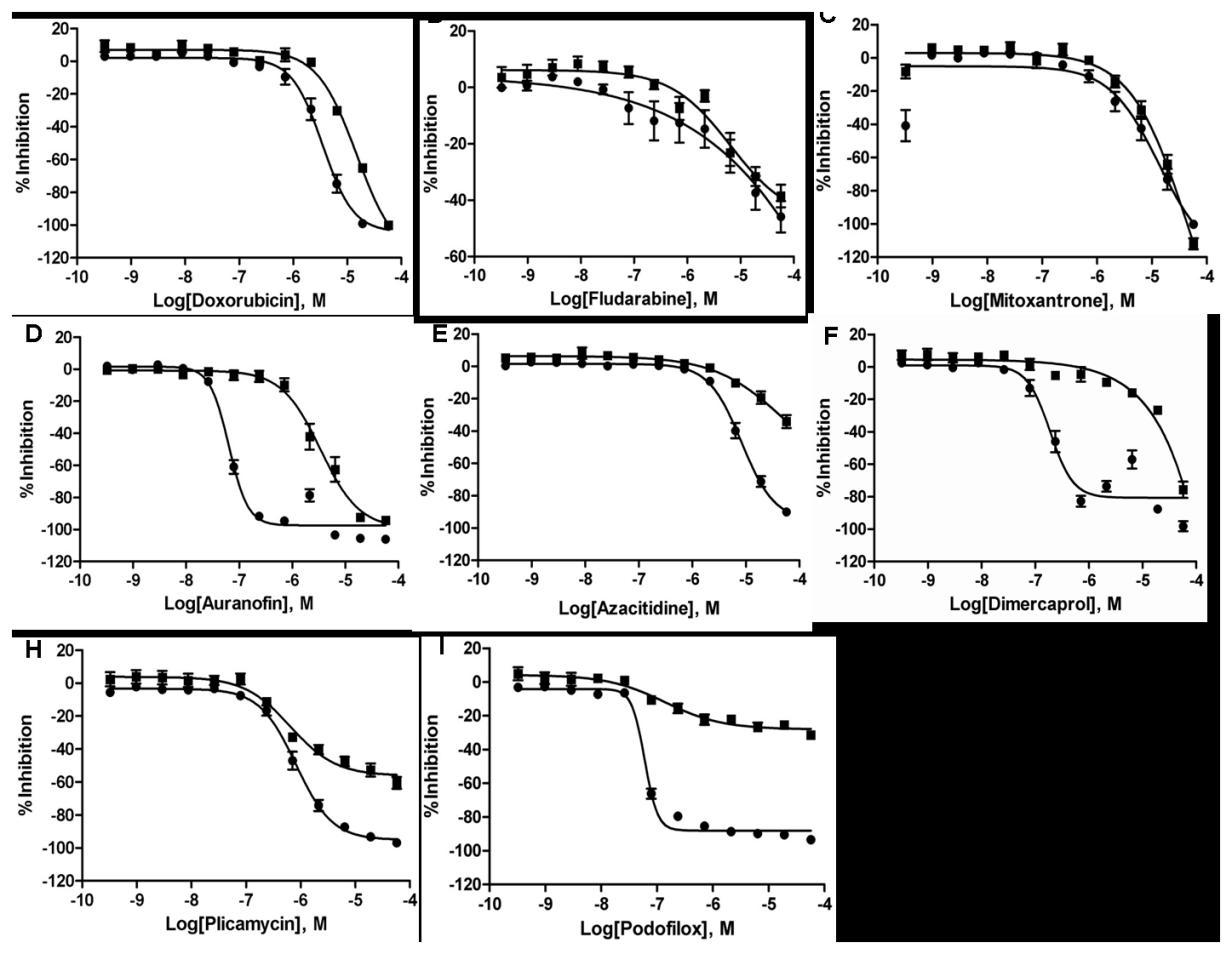
Concentration-response curves of five selected drugs, classic chemotherapy drug doxorubicin, and CLL frontline drugs (fludarabine and mitoxantrone) in the CLL cytotoxicity assay. Each CRC represents the average response of multiple samples testing with error bars demonstrating the SD. Data was normalized to DMSO basal (0%) and control compound doxorubicin (-100%). Activity against cells from 7 CLL patients (●) and 2 normal donors (■). IC_50_s are: Doxorubicin: CLL = 4.5 µM, normal = 14.4 µM; Fludarabine: CLL = 12.2 µM, normal = 9.4 µM; Mitoxantrone: CLL = 7.2 µM, normal = 7.0 µM; Auranofin: CLL = 0.07 µM, normal = 2.05 µM; Azacitidine: CLL = 8.1 µM, normal = 15.9 µM; Dimercaprol: CLL = 0.38 µM, normal = 19.4 µM; Activity for Plicamycin: CLL = 0.75 µM, normal = 0.84 µM; Activity for Podofilox: CLL = 0.06 µM, normal = 0.15 µM.

The five selective agents along with the classic chemotherapy drugs doxorubicin, fludarabine, and mitoxantrone, were further evaluated in the cell viability assay using fresh lymphocyte samples obtained from seven CLL patients and two normal donors. Data is shown in Figure **5**.

As expected doxorubicin was highly cytotoxic, but non-selective in that it killed CLL cells and normal lymphocytes with equal potency (potency shift of 3-fold: CLL IC_50_ = 4.5 µM, normal lymphocytes IC_50_ = 14.4 µM, Figure **5A**). Fludarabine and mitoxantrone also showed comparable cell killing against CLL cells and normal lymphocytes with potency at 12 µM and 6 µM, respectively (Figure 5B and 5C). In contrast, auranofin and dimercaprol exhibited 30- and 50-fold shifts in potency between CLL and normal donor samples respectively, demonstrating a possible therapeutic window for CLL patients (Figures 5D and 5F).

Although the potency shifts for azacitidine, plicamycin and podofilox were only about 3-fold, all of these compounds possessed differential cytotoxicity as demonstrated by their considerable differences in efficacy (over 40% difference at 19 µM and 57 µM, the two highest testing concentrations) between CLL and unaffected donor samples, suggesting some degree of specificity for the leukemic cells (Figures **5E, 5H** and **5I**). Podofilox showed potent cytotoxicity (> 60% efficacy) at concentrations as low as 80 nM with almost no cytotoxicity on lymphocytes derived from unaffected donors (Figure **5I**).

### Effect of five selected drugs on caspase activity

**Figure 6:**
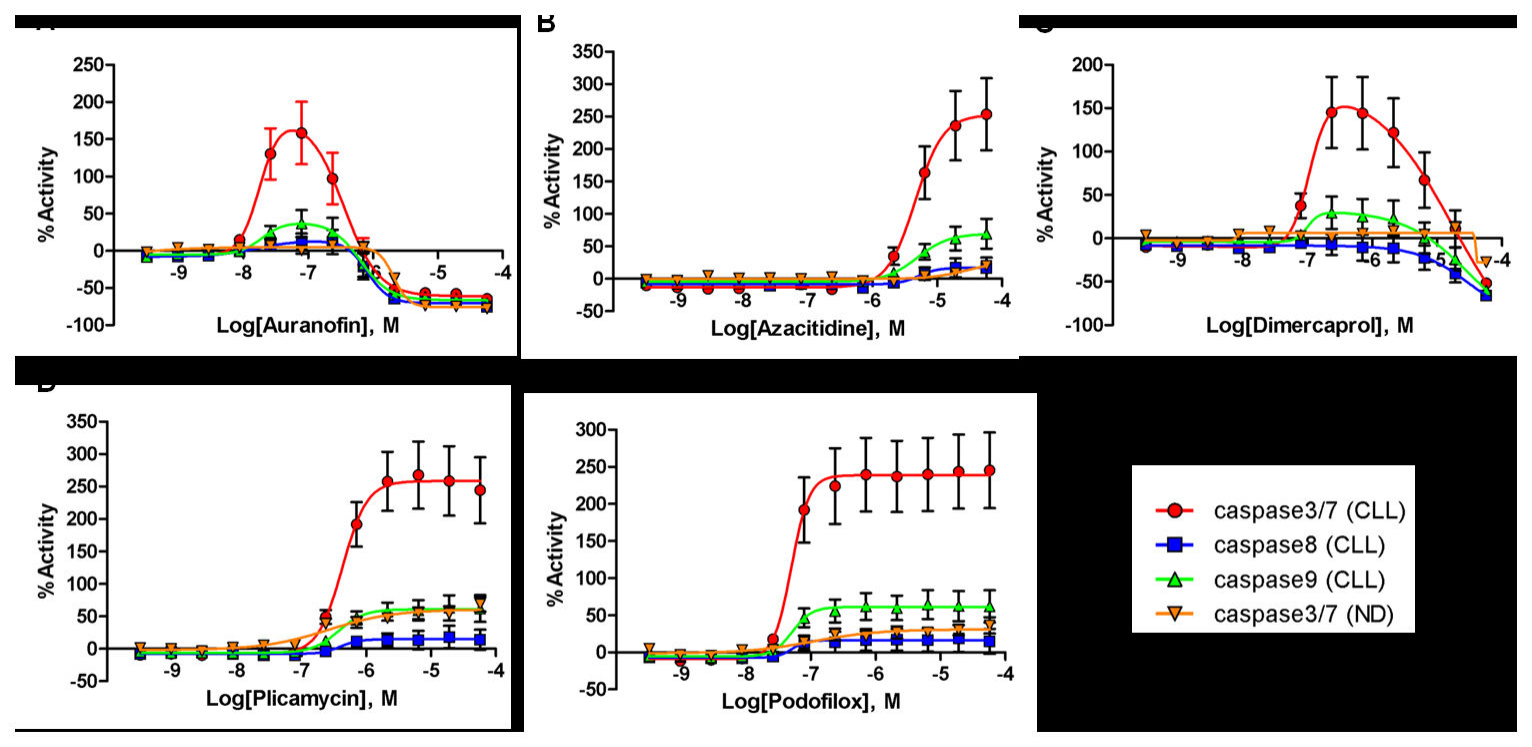
Caspase-3/7, caspase-8 and caspase-9 activation in response to the five selected agents in samples from CLL patients and normal donors. Each CRC represents the average response of multiple samples testing with error bars demonstrating the SD. The results are shown in %activity of caspase activity in drug treated cells compared to the corresponding DMSO treated controls (0% = DMSO treated control, 100% = 2-fold caspase activity induction of DMSO control).

To determine the effect of these five selected compounds on apoptosis, caspase-3/7, caspase-8 and caspase-9 activity was measured in CLL samples after compound treatment at 24 hours. All five compounds activated caspase-3/7 but not caspase-8 and 9 (Figure **6**), consistent with triggering the intrinsic apoptotic pathway. Auranofin, podofilox and dimercaprol were the three most potent stimulators of caspase-3/7 activity, with an EC_50_ value below 100 nM (Figures **6A, 6E** and **6C**), followed by plicamycin (0.43 µM, Figure **6D**) and azacitidine (5.1 µM, Figure **6B**). Efficacies in the caspase assay were calculated relative to DMSO basal and the level of the stimulation of caspase-3/7 activity varied between these five compounds. Noteworthy all five compounds did not show significant caspase-3/7 activation in lymphocytes from normal donors. Auranofin and dimercaprol were observed to have bell-shaped curves, likely due to the significant cytotoxicity which induced cell death more rapidly than the other agents at the higher testing concentrations.

### Known CLL agents or combination chemotherapy agents in CLL screening

In addition to the positive control (doxorubicin), we were also interested in the activity outcomes of known CLL drugs in the *in vitro* cell viability assay. There are a few drugs in the NPC drug library that are known frontline or combination therapy drugs in the clinical use for the treatment of CLL including fludarabine (Fludara®), chlorambucil, bendamustine, mitoxantrone, vincristine and vinblastine. In general, these drugs usually showed increased potency and efficacy with longer incubation times (Table **S2**), but all of these drugs demonstrated some degree of cytotoxicity at the 24hr time point, which was the final incubation condition adopted for the 1536-format high-throughput screening. Vincristine and its chemical analogue vinblastine, two chemotherapeutic drugs known as vinca alkaloids [25], showed approximately equal potency in our CLL drug screening with IC_50_’s around 0.24 to 0.58 µM (Table **S1**). Mitoxantrone is a type II topoisomerase inhibitor; it disrupts DNA synthesis and DNA repair in both healthy cells and cancer cells [26]. In our drug screening, mitoxantrone showed notable cell killing with average IC_50_ around 5.4 µM (Table **S1**). Fludarabine, a purine analog which is known to inhibit DNA synthesis by interfering with ribonucleotide reductase and DNA polymerase [27], along with the other two agents chlorambucil and bendamustine, nitrogen-containing alkylating agents derived from mustard that cause intra-strand and inter-strand cross-links between DNA bases [28], showed varied potencies ranging from 12 to 30 µM approximately (Table **S1**).

## Discussion

In the present study, we have identified many drugs that showed significant cytotoxicity to CLL primary cells by screening a comprehensive collection of approximately 2,800 small molecules that are either approved for human use or are currently in clinical trials. To our knowledge, this is the first study to examine such a large set of clinically approved compounds to identify novel pharmaceutical agents which could be used in the treatment of CLL. The cell viability assay employed a qHTS format so that potency values were obtained for every compound and the assay was found to be easy to implement and highly robust. This format allows one to compare pharmacological parameters, for example potency and efficacy values. Using this assay we also compared the cytotoxic effects of drugs on CLL cell samples to cells from unaffected normal donors.

Our assay identified over 100 FDA approved compounds with anti-CLL activity including, as expected chemotherapeutic agents used in this disease such as fludarabine, chlorambucil, bendamustine, mitoxantrone and vincristine. Approximately 95% of the CLL consensus actives identified from the primary screening were not selective against unaffected donor cells, indicating that our assay does not reflect a “therapeutic window” for these drugs currently in clinical use. Remarkably, we identified 5 compounds that were selectively toxic for CLL cells in that the IC50 against normal cells was >5-fold the IC50 against the tumor cells. These results were confirmed with additional compound samples and fresh lymphocytes obtained from both of the two unaffected and seven CLL donor samples which was collected within hours of apheresis. Among the five selective CLL cytotoxic agents we found that auranofin, dimercaprol, plicamycin and podofilox were all very potent drugs with IC_50_ < 1 µM, while azacitidine was moderately active with IC_50_ ~ 8.1 µM.

Auranofin is a gold containing drug approved and used throughout the EU and USA to treat rheumatoid arthritis since the early 1980s [29]. Auranofin is known to inhibit the levels of pro-inflammatory cytokines such as interleukin 1 beta (IL-1β) and tumor necrosis factor (TNF) α by inhibiting the transcriptional activity of nuclear factor kappa light chain enhancer of activated B cells NFkB. It has also been shown to block the interleukin 6 (IL-6) signaling by inhibiting Janus kinase signal transducer and activator of transcription 3 JAK1-STAT3 signaling. Auranofin also exhibits antineoplastic activity and inhibits DNA synthesis. Although the precise mechanism underlying this antineoplastic effect is not known, one possibility is that this arises from inhibition of the thioredoxin reductase/thioredoxin system, as it has been found that auranofin, along with another organic gold compound aurothioglucose, can strongly inhibit human thioredoxin reductase in its NADPH-reduced form [30]. Auranofin is a potent cytotoxic agent with an IC_50_ of 70 nM (average value from 7 patient samples) and selective for leukemia cells with a greater than 30-fold separation in cytotoxicity compared to normal cells. Moreover, auranofin has been found to be effective in increasing the life span of mice inoculated with the lymphocytic leukemia P388 [31]. Based on additional in-vitro work that supports its activity [32,33], auranofin has been advanced into a Phase II clinical study in CLL [34].

Azacitidine is a nucleoside analogue of cytidine that specifically inhibits DNA methylation by trapping DNA methyltransferases [35]. It is currently approved for the treatment of myelodysplastic syndrome (MDS) [36]. Azacitidine is thought to exert its antineoplastic effects in part by causing hypomethylation of DNA and targeting 'epigenetic' gene silencing, a mechanism that is exploited by cancer cells to inhibit the expression of genes that counteract the malignant phenotype [37]. It has also been used in the treatment of acute myelogenous leukemia AML [38]. Our data suggested that azacitidine is a selective agent killing CLL malignant cells with over 40% decreased efficacy to normal B cells. It is interesting to note that azacitidine had been advanced to phase II clinical trial for the treatment of CLL [39] in Texas M.D. Anderson Cancer Center in collaboration with Celgene Inc, but the trial was prematurely discontinued because of lack of response and slow accrual [40].

Dimercaprol was originally developed as an antidote to combat the effects of the blister gas lewisite 60 years ago [41]. It has been commonly used as a chelating agent in arsenic, mercury, gold, lead, and other toxic metal poisoning [42]. In addition, in the past dimercaprol has been used to treat patients with Wilson's disease (hepatolenticular degeneration), which is a genetic disorder resulting in excess copper accumulation, primarily in the brain and liver [43]. In our CLL assay, this compound showed consistent cytotoxicity to patient cells with an average IC_50_ value of 0.38 µM and was 50-fold selective to normal donor cells. Dimercaprol reportedly is quite toxic with a low safety margin and a tendency to redistribute arsenic to other organs such as brain and testes. Other serious side effects include nephrotoxicity and hypertension. In addition, it cannot be given orally but has to be administered through painful intramuscular injections [44]. Given of the undesirable side effect profile, dimercaprol is now infrequently used.

Podofilox, also called podophyllotoxin, is one of the well-known naturally occurring antimitotic agents that inhibit microtubule assembly [45]. It used as a topical drug to treat certain types of warts on the outer skin of the genital areas. Podofilox is also the pharmacological precursor for the important semisynthetic derivatives teniposide, and etoposide, DNA topoisomerase II inhibitors that are commonly used as cancer chemotherapeutic agents in multiple indications. Unlike etoposide and teniposide, podofilox’s anticancer property can be attributed to the inhibition of microtubule polymerization which leads to mitosis failure and cell cycle arrest [46,47]. In our CLL cell viability assay, this compound yielded a potency of 60 nM with CLL patient cells and approximately 40% decreased efficacy against B-cells from normal donors at the testing concentration of 1.7 µM.

Plicamycin, also referred to as mithramycin, is an antineoplastic antibiotic isolated from *Streptomyces* *plicatus* . It binds to DNA and inhibits DNA, RNA, and protein synthesis [48]. It was used in the treatment of testicular carcinomas and acute myelogenous leukemia [49,50]. In a pilot clinical study using plicamycin and alpha-interferon as the combination therapy, plicamycin appeared to add efficacy to interferon in the stabilization of accelerated phase of chronic myeloid leukemia (CML). As well, there are data suggesting that plicamycin can induce differentiation of blastic CML cells, and anecdotal clinical responses have been reported [51]. Plicamycin was one of the most potent compounds in our CLL screening with an IC_50_ of 0.75 µM and selectively killed CLL malignant cells with over 40% decreased efficacy to normal B cells.

On the other hand, most of the first line CLL drugs only showed moderate or weak activity in our cell viability assay. One possibility for the lower potency and efficacy of these known CLL drugs is because these compounds are commonly used in combination during cancer therapy, so it is likely compounds have synergistic activity. For example, fludarabine is mainly used in various combinations with cyclophosphamide, mitoxantrone, dexamethasone and rituximab in the treatment of indolent non-Hodgkins lymphomas [52]. Fludarabine, cyclophosphamide, and mitoxantrone (FCM) combination therapy results in a high response rate in previously treated patients with CLL [53]. Expanding our current drug screening to examine combinations in the cell viability assays will be of interest.

In addition to small molecule chemotherapy agents, using targeted drug therapy is another attractive approach and several monoclonal antibodies are being used in treating CLL including rituximab (Rituxan, directed against CD20), alemtuzumab (Campath, directed against CD52) and ofatumumab (Arzerra, directed against CD20). CLL cells express a variety of proteins on the cell surface which distinguishes these cells from normal cells, targeted drugs are designed to attack specific vulnerabilities in these cancer cells. However, the NPC drug library contains only low molecular weight compounds and hence the in vitro activity of antibodies against the primary CLL patient cells was not measured in the assay described here.

In summary, our CLL assay provided results pertaining to new information on approved drugs as CLL cytotoxic agents which suggests novel applications and possible mechanisms of actions for these compounds. This approach to screening a clinically approved drug library can be used to screen for drug activity in other cancer cell lines, primary patient cells from various hematologic malignancies, and different tumor types. Results from phenotypic assays can be difficult to translate into effective therapeutics. However, our results suggest that phenotypic assays employing primary disease cells in combination with a high-value compound library containing known drugs can enable the rapid translation of discovery efforts into therapeutics. In the CLL assay, primary disease cells are used and the assay endpoint is directly related to the desired therapeutic endpoint (e.g. selective toxicity to primary CLL cells). It is worth noting that drug toxicity is a common reason for failure of a drug candidate in clinical trials, however screening existing drugs that have already overcome this barrier for new therapeutic indications could provide a faster path for new patient therapies. Therefore, identification of anticancer effects of existing FDA approved compounds with known toxicity profiles provides an accelerated path to clinical trials to directly test their efficacy in new indications. This general approach could lead to drug repurposing and accelerate clinical development of compounds with well established toxicity profiles for many types of malignances.

## Supporting Information

Figure S1 **Activity outcomes of the 2816 compounds in NPC drug collection tested in the cell viability assays using primary cells obtained from 6 CLL patients.**
(TIF)

Table S1 **Compound potency, efficacy, curve class and therapeutic category for 41 selected compounds in the confirmation assay.**
(TIF)

Table S2 **Compound potency and efficacy at different time points in the CLL viability assay.**
(TIF)
