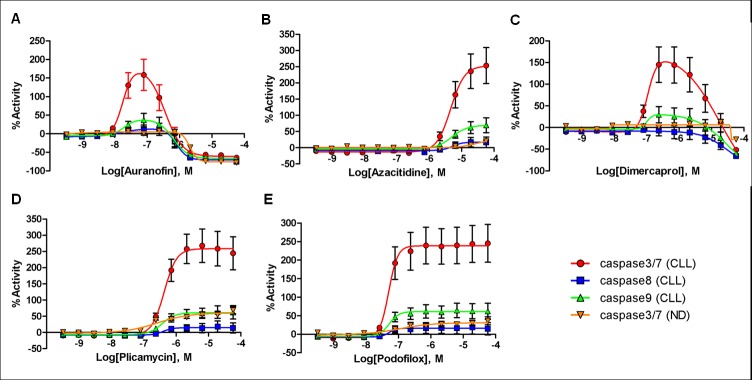# Correction: Identification of Therapeutic Candidates for Chronic Lymphocytic Leukemia from a Library of Approved Drugs

**DOI:** 10.1371/annotation/e2536fcb-3ab3-44a0-8eab-91aaeb8e49b6

**Published:** 2013-11-01

**Authors:** Min Shen, Yaqin Zhang, Nakhle Saba, Christopher P. Austin, Adrian Wiestner, Douglas S. Auld

There were errors in Figures 2-6. Correct versions of these figures are available below.

Figure 2: 

**Figure:**
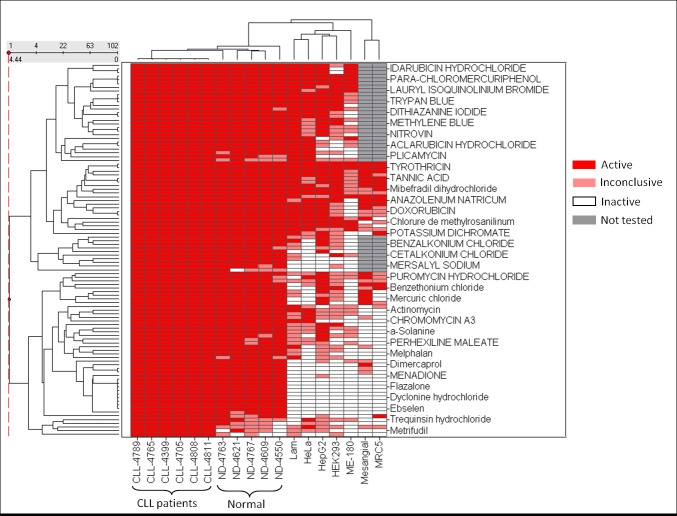


Figure 3: 

**Figure:**
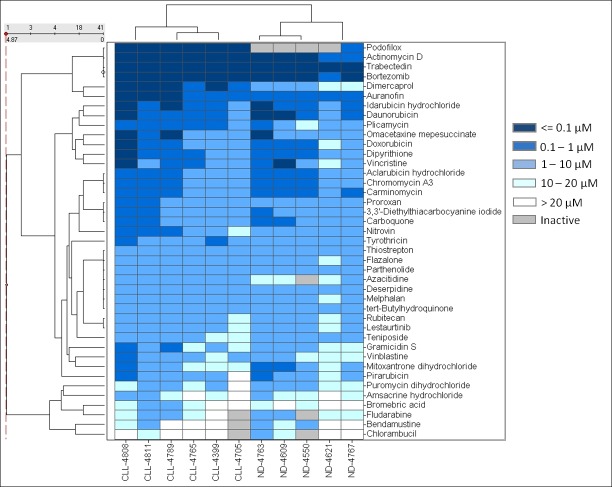


Figure 4: 

**Figure:**
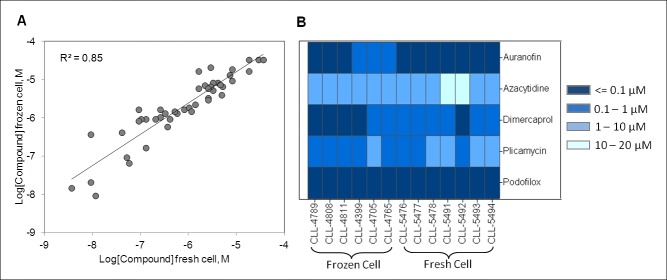


Figure 5: 

**Figure:**
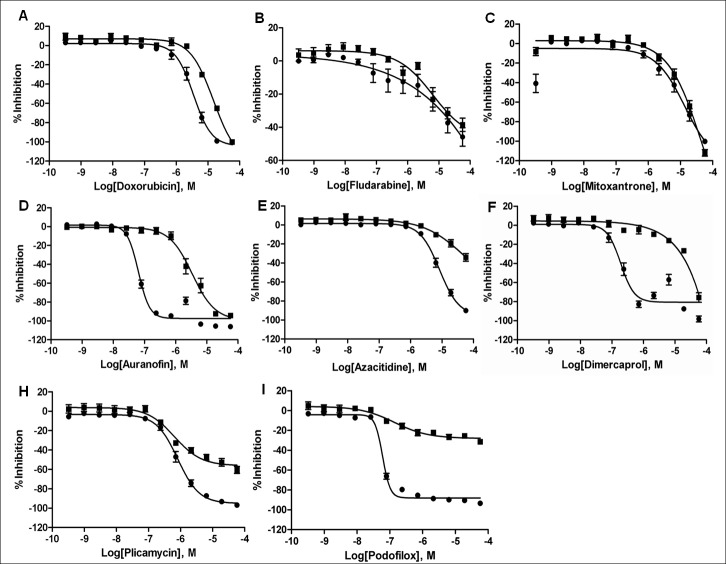


Figure 6: 

**Figure:**