# Supplementary figures and images for: Identification of Therapeutic Candidates for Chronic Lymphocytic Leukemia from a Library of Approved Drugs
Source: PLoS One. 2013 Sep 20;8(9):e75252. doi: 10.1371/journal.pone.0075252 (PMC3779154; doi:10.1371/journal.pone.0075252)

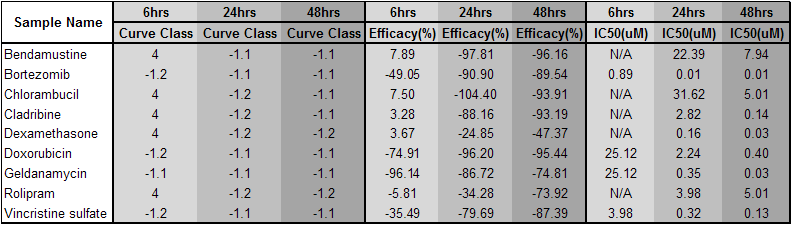

Supplement: Table S2 — Compound potency and efficacy at different time points in the CLL viability assay. (TIF) [file pone.0075252.s003.tif]
